# The MicroRNA-Based Strategies to Combat Cancer Chemoresistance *via* Regulating Autophagy

**DOI:** 10.3389/fonc.2022.841625

**Published:** 2022-02-08

**Authors:** Yuhe Lei, Lei Chen, Junshan Liu, Yinqin Zhong, Lijuan Deng

**Affiliations:** ^1^Shenzhen Hospital of Guangzhou University of Chinese Medicine, Shenzhen, China; ^2^School of Traditional Chinese Medicine, Southern Medical University, Guangzhou, China; ^3^Department of Pharmacy, Zhujiang Hospital, Southern Medical University, Guangzhou, China; ^4^Formula-Pattern Research Center, School of Traditional Chinese Medicine, Jinan University, Guangzhou, China

**Keywords:** microRNA, chemoresistance, autophagy, cancer therapy, non-coding RNA

## Abstract

Chemoresistance frequently occurs in cancer treatment, which results in chemotherapy failure and is one of the most leading causes of cancer-related death worldwide. Understanding the mechanism of chemoresistance and exploring strategies to overcome chemoresistance have become an urgent need. Autophagy is a highly conserved self-degraded process in cells. The dual roles of autophagy (pro-death or pro-survival) have been implicated in cancers and chemotherapy. MicroRNA (miRNA) is a class of small non-coding molecules that regulate autophagy at the post-transcriptional level in cancer cells. The association between miRNAs and autophagy in cancer chemoresistance has been emphasized. In this review, we focus on the dual roles of miRNA-mediated autophagy in facilitating or combating chemoresistance, aiming to shed lights on the potential role of miRNAs as targets to overcome chemoresistance.

## Introduction

Cancers with local organ invasion and distant metastasis often require systemic chemotherapy. Despite the newly developed therapeutic interventions such as immunotherapy, chemotherapy is still the most commonly applied treatment modality (1). In recent years, neoadjuvant chemotherapy has been included in the treatment guidelines of various solid tumors (2). However, after benefiting from the initial chemotherapeutic treatment, most patients will inevitably suffer from cancer relapse because of acquiring chemoresistance (3). Chemoresistance, a major cause of treatment failure and high mortality, remains a big challenge in clinics. Acquired drug resistance occurs after long-term chemotherapy, followed by devastating outcome (4), whereas intrinsic drug resistance exists without exposure to therapeutic drugs (5). It is reported that chemoresistance is responsible for more than ninety percent of cancer-related mortality (6). For instance, it has been documented that almost half of the patients diagnosed with metastatic colorectal cancer are resistant to 5-FU-based chemotherapy and their five-year survival rate is only slightly over 12% (7). Hence, there is an urgent need to elucidate the mechanism of chemoresistance and explore novel treatment strategies. After decades of works, several strategies to reverse chemoresistance have been proposed, including inhibition of P-glycoprotein (P-gp), combinational therapy, dosage enhancement, tumor microenvironment modulation and so on (8). Up to now, four generations of drug resistance reversal agents have been developed. The first generation of P-gp inhibitors such as verapamil and cyclosporin A can sensitize tumor to chemotherapeutic drugs only *in vitro* but not *in vivo* (9). The second generation of P-gp inhibitors such as S9788 and PSC833 also can’t be used clinically because it can inhibit cytochrome P4503A4 to bring about unpredictable toxicity and side effects (10). The third generation including tariquidar, laniquidar, zosuquidar and fourth generation including curcumin, andreia, tangeretin are under laboratory or clinical investigation and expected to be clinically used in the future (11). In addition, immunotherapy and targeted therapy are commonly used in clinic after chemoresistance occurs (12). Among these strategies, targeting autophagy to combat chemoresistance is gradually coming into sight.

Autophagy is an evolutionarily conserved process in which long-lived proteins, damaged organelles, or other cytoplasmic components are degraded and recycled to maintain energy homeostasis of cells (13). In 2016, Yoshinori Ohsumi was awarded the Nobel Prize for his contributions in elucidating the mechanism of autophagy, making autophagy a highlighted focus (14). Dysregulation of autophagy is involved in various pathological events such as cardiovascular disease (15), neurological disease (16), endocrine disorder (17), and especially cancers (18). Autophagy occurs frequently during chemotherapy, acting as either a pro-death or pro-survival process (19). The dual roles of autophagy in multi-drug resistance (MDR) have been described in our previously published review (13). On one hand, autophagy protects cancer cells from chemotherapeutic drugs to mediate drug resistance by eliminating damaged organelles and recycling degradation products. On the other hand, excessive autophagy can kill MDR cancer cells in which apoptosis pathways are inactive. Therefore, it is well recognized that autophagy is involved in chemoresistance in various types of cancers (20). The role of autophagy in chemoresistance is paradoxical and context-dependent, which needs comprehensive and systematic investigation.

MicroRNAs (miRNAs) are a class of small non-coding RNA with 19-25 nucleotides. They regulate gene expression by binding to the 3’-untranslated region (UTR) of target mRNAs to inhibit mRNA translation or facilitate mRNA degradation (21). Abnormal expression of miRNAs has been implicated in regulating cell proliferation, apoptosis, metastasis, migration, autophagy, and drug resistance in a large number of cancer types (22). Accumulating evidence indicated that miRNAs target some of the molecules in autophagic pathway thus resulting in chemoresistance or chemosensitivity during chemotherapy. Therefore, miRNAs could be promising targets for reversal of chemoresistance (23). Currently, miRNA-based therapies have been proposed. MiRNA mimics, miRNA sponges, anti-miRNA oligonucleotides, and small molecule inhibitors are promising strategies to modulate miRNAs (24). Miravirsen, the first miRNA-targeted drug, has been successfully tested in clinical Phase II trials for the treatment of hepatitis C (25). Miravirsen is a locked nucleic acid (LNA)-based antisense oligonucleotide targeting miR-122 (26). In the field of oncotherapy, MRX34, a liposomal miR-34a mimic, is the most advanced miRNA drug, which was designed to deliver miR-34a mimic to cancer cells for the treatment of several solid tumors (27). Additionally, novel miRNA-based drugs are being developed for the treatment of atherosclerosis (anti-miR-33a/b) (28), chronic heart failure (anti-miR-208, anti-miR-195) (29, 30), and other diseases.

In this review, we discussed the correlation between miRNAs and autophagy in chemoresistance/chemosensitivity, illustrated the current interventions targeting miRNA/autophagy axis to combat chemoresistance, aiming to provide novel insights from the perspective of miRNA-mediated autophagy for promoting chemotherapeutic efficacy.

## Autophagy in Cancers

Autophagy is initiated by the formation of double-membraned autophagic vesicles (AV) in response to a range of cellular stresses, including nutrient deprivation, hypoxia, organelle damage, and accumulation of reactive oxygen species (ROS) (31). The critical roles of autophagy in cell death, cell survival, metabolic adaptation, embryonic differentiation, immune surveillance and other biological processes have been verified (32). Therefore, dysregulation of autophagy has been implicated in various diseases such as Alzheimer’s disease, aging, microorganism infection, and multiple forms of cancers (33).

There are three types of autophagy, namely macroautophagy, microautophagy, and chaperone-mediated autophagy (34). Hereafter autophagy refers to macroautophagy, which is well understood and the mechanisms are established. In addition to general autophagy which functions in bulk degradation of cytoplasmic material, there exists selective autophagy targeting specific proteins or organelles such as mitochondria, endoplasmic reticulum (ER), bacteria, ribosomes, and ferritin (35). It is well accepted that autophagy is a multistep process involving approximately 30 autophagy-related genes (*Atgs*), that encode proteins executing the initiation of phagophore, AV maturation, and lysosomal fusion (36).

Mammalian target of rapamycin (mTOR) is at the upstream position of the autophagic process. mTOR consists of two distinct multiprotein complexes: mTORC1 and mTORC2 (37). As an environmental sensor, mTOR responds to intracellular and extracellular stressful conditions such as hypoxia, nutrient deprivation, or drug treatment (38). mTOR actively phosphorylates ATG, leading to the inhibition of autophagy under nutrient-rich conditions. In the case of nutrient deprivation, mTOR is inactivated and can no longer phosphorylate and inhibit the Unc-51-like kinase (ULK) complex, which consists of ULK family kinase, focal adhesion kinase interacting protein 200 kDa (FIP200), and ATG13. Dephosphorylated ULK1 dissociates from the mTOR complex and becomes active to trigger autophagosome membrane nucleation (39, 40). In addition to mTOR, 5’-AMP activated protein kinase (AMPK) also acts as a master regulator of energy stress to participate in the activation of ULK complex (41). Once activated, the ULK complex localizes to the phagophore and activates the Beclin1-Vacuolar protein sorting associated protein 34 (VPS34) complex, which contains VPS34, VPS15, Beclin1, and ATG14L (42). The VPS34 (a class III phosphatidylinositol 3-kinase, PI3K) complex generates phosphatidylinositol 3-phosphate (PI3P)-rich membranes most commonly derived from endoplasmic reticulum and Golgi complex (43). Elongation of phagophore membrane relies on two ubiquitin-like conjugation systems, the E1-like enzyme ATG7 and E2-like enzyme ATG10, which conjugate ATG5 to ATG12 (44). The E3 like enzyme ATG5-ATG12-ATG6L1 complex together with ATG7-ATG3 complex conjugate microtubule-associated protein 1 light chain 3 (LC3, ATG8) family members to phosphatidylethanolamine (PE) (45). The conversion of pro-LC3 to the active cytosolic isoform LC3-I requires the ATG4 family of cysteine proteases (46). Next, LC3-I is conjugated to PE to generate LC3-II, which is regarded as a key step of specific substrate recognition for selective degradation, therefore constructing cargo-loaded autophagosomes (47). In addition to serving as a marker for autophagosome, LC3 also acts as a docking site for cargo adaptors that bring autophagic cargo to the AVs. These adaptors such as SQSTM1 (p62) and neighbor of BRCA1 (NBR1) directly bind to proteins and organelles marked for autophagic degradation through NIX and FAM134B (48). Then, the double-membrane autophagosomes are degraded by fusing with lysosomes to form autolysosomes, that are regulated by Rab GTPases, SNARE, and HOPS complex (49, 50). During this process, the outer autophagosomal membrane is cleaved by ATG4, while the LC3-PE-conjugated inner membrane and the cytoplasmic contents were broken down by lysosomal proteases, thus recycling amino acids and other macromolecular building blocks (32).

The dual roles of autophagy in cancers have been largely demonstrated. Although autophagy may limit tumorigenesis in the earliest stage, accumulating evidence indicate that antophagy inhibition displays anti-proliferative effects in established cancers since antophagy can help cancer cells cope with hypoxia, nutrient deprivation or other cellular stresses (51). The basal level of autophagy plays a protective role against cancer through eliminating damaged organelles and proteins to maintain cellular homeostasis in normal cells (52). The abnormal autophagy contributes to the development of cancers. The first mouse model with deletion of autophagy gene was established to study the role of autophagy in tumorigenesis, and the results indicated that the deletion of *BECN1* (gene symbol of Beclin1) increased the rate of spontaneous tumor formation compared with *BECN1* wild-type (53). Depletion of the *BECN1* is also observed in human breast, prostate, and ovarian cancers (54). Additionally, bax-interacting factor 1 (BIF-1) and UV radiation resistance-associated gene protein (UVRAG), which is related to Beclin1, was found to be absent or mutated in variety of cancer types (55, 56). However, a high basal-level of autophagy is observed in multiple established cancers, acting as a protective mechanism towards nutrient-stressed conditions (57). For example, in a *Kras*-driven lung cancer model, tumor cell growth and survival requires autophagy which plays a vital role in maintaining mitochondrial function (58). As a consequence, inhibition of cytoprotective autophagy in these cancers may result in tumor suppression (59). Additionally, a large body of literature has emerged and elucidated the role of autophagy induction to enable survival of cancer cells following chemotherapy or radiotherapy, indicating that autophagy is a key drug resistance mechanism in various cancer types (60).

## Dual Roles of Autophagy in Chemoresistance

Chemoresistance is a major cause of treatment failure, cancer relapse, and cancer metastasis (3). Drug resistance can be classified as resistance to either a single agent or multiple drugs with different structures and mechanisms of action (referring to MDR) (61). Mechanisms of cancer chemoresistance mainly include the following categories: (1) increased drug efflux by membrane transporters particularly ABC transporters (62), (2) reduced drug uptake by influx transporters such as solute carriers (63), (3) alterations in tumor microenvironment (TME), through secretion of multiple growth factors, chemokines, and cytokines by stromal and immune cells (64), (4) cancer stem cells, a class of tumor-triggering cells able to self-renew (65), (5) excessive DNA repair, which makes cancer cell survive and become tolerant to chemotherapeutic agents (66), (6) boosting drug metabolism mediated by glutathione S-transferase and cytochrome P450 enzymes (67, 68), (7) mutation in cancer-related genes including gain of function in oncogenes and loss of function in tumor suppressor genes (69), and (8) elevating adaptability by epigenetic and/or miRNA regulation (1).

The relationship between chemoresistance and autophagy has been studied for decades. It is known that resistance of cancer cells to chemotherapeutic agents is inevitable following prolonged exposure to drugs. This phenomenon may be partly mediated by induction of autophagy as a protective mechanism to cope with pressures during treatment (70). The autophagy triggered by chemotherapeutic drugs such as paclitaxel, epirubicin, or tamoxifen facilitates resistance of cancer cells to corresponding or multiple drugs (71–73). Various autophagy regulators and signaling pathways were confirmed to participate in this process. The mechanisms of metabolic-induced and therapeutic stress-induced autophagy might overlap in cancers. After chemotherapy is applied, nutrient and energy stress is amplified to increase autophagic flux. For example, after treatment with mTOR inhibitors, the transcription factor EB (TFEB)/transcription factor E3 (TFE3)/melanocyte inducing transcription factor (MITF) family can no longer be phosphorylated and translocate to nucleus, therefore activating transcription of the CLEAR network of genes to affect lysosome and autophagy (74). Another research demonstrated that the expression of S100A8 which is necessary for Beclin1-PI3KC3 complex formation is elevated to promote autophagy after adriamycin and vincristine treatment, contributing to drug resistance in leukemic cells (75). Moreover, ATG family members (76), bromodomain containing 4 (BRD4) (77), p53 (78), and ER stress-related genes (79) are also important factors involved in cytoprotective autophagy to mediate chemoresistance. Therefore, inhibition of such autophagy can re-sensitize resistant cancer cells to chemotherapeutic drugs. In recent years, the combination strategies of chemotherapeutic drugs and autophagy inhibitors have been proposed. Abundant basic and clinical research is ongoing. It is well established that genetic silencing of ATGs such as ATG5, ATG7, and Beclin1 blocks autophagy to sensitize drug resistant cells to therapeutic agents (80, 81). Chloroquine (CQ) and hydroxychloroquine (HCQ), which are clinically used for malaria treatment, are potent autophagy inhibitors through destroying lysosomes to prevent autophagosome degradation (82). Previous research has revealed that inhibition of autophagy by CQ sensitize vincristine-resistant gastric adenocarcinoma (83), epirubicin-resistant triple-negative breast cancer (84), sorafenib-resistant hepatocellular carcinoma (HCC) (85), cisplatin-resistant hypopharyngeal carcinoma (86), 5-fluorouracil-resistant gallbladder carcinoma (87) to chemotherapeutics. HCQ has been repurposed in numerous clinical trials either as a single agent or combined with therapeutic agents, some of which are in phase II studies (88). Lys05, a water-soluble analog of HCQ, displays stronger anticancer properties than HCQ both *in vitro* and *in vivo* (89). It can improve the efficiency of BRAF inhibitor against glioblastoma (90). Other autophagy-targeted compounds that are promising in combating chemoresistance include wogonin (91), SAR405 (92), tioconazole (93), 3-methyladenine (3-MA) (94) and others.

Paradoxically, while autophagy mainly acts as a pro-survival mechanism, excessive autophagy leads to a caspase-independent cell death called “type II programmed cell death” or “autophagic cell death”, which differs from apoptosis (95). In consequence, activation of such autophagy confers lethal effect on drug resistant cancer cells (96). Numerous studies have focused on identifying the novel agents that can effectively kill apoptosis-deficient cancer cells by inducing autophagic cell death. Since AKT/mTOR is the vital negative regulator of autophagy, the AKT/mTOR-associated autophagic cell death has gained a lot of attention. As a dual PI3K and mTOR inhibitor, NVP-BEZ235 was reported to sensitize osteosarcoma and urothelial cancer cells to cisplatin by activating autophagic flux independent of apoptosis (97, 98). Meanwhile, NVP-BEZ235 can also combat resistance to temozolomide and doxorubicin in glioma and neuroblastoma cells respectively (99, 100). Similarly, the Ganoderma microsporum immunomodulatory (GMI) protein targets AKT-mTOR-p70S6K pathway to reverse multidrug resistance by inducing pro-death autophagy in lung cancer (101). In addition to AKT/mTOR signaling pathway, the JNK activation and MCT1 inhibition also contributes to autophagic cell death, suggesting the possible autophagy-related targets to overcome chemoresistance (102, 103). Therefore it can be seen that autophagy demonstrates a role of pro-survival or pro-death to promote or suppress tumor growth, as well as mediate or combat chemoresistance. Inhibition of cytoprotective autophagy may enhance the sensitivity of cancer cells to chemotherapeutic agents, whereas induction of autophagic cell death can be used as an alternative cell death mechanism when the cells fail to undergo apoptosis. It is convinced that the role of autophagy is context- and tumor type-dependent, therefore clarifying the relationship between autophagy and chemoresistance is urgent and critical for improving the efficacy of chemotherapy.

## MiRNAs Combat Chemoresistance by Regulating Autophagy

MiRNAs are a class of small non-coding single-stranded RNA molecules with 19-25 nucleotides. They play fundamental roles in multiple biological processes through binding to the 3’-UTR of target mRNAs to accelerate mRNA degradation or terminate translation (104). MiRNAs are evolutionary conserved and found in a wide range of organisms (105). It is reported that more than 60% of human genes contain potential miRNA binding sites and approximately 10-40% of mRNAs are regulated by miRNAs (106). By post-transcriptional gene silencing, miRNAs regulate various cellular pathways including cell growth, differentiation, apoptosis, and homeostasis (107). MiRNA genes exist in both intergenic and intronic regions (108). They are transcribed into primary miRNA (pri-miRNA) with 5’ cap and a 3’ poly-A tail by RNA polymerase II (109). Meanwhile, RNA polymerase III is required for the transcription of some particular miRNAs (110). Following transcription, pri-miRNAs are processed in the nucleus by a core microprocessor complex including RNase III enzyme Drosha and its cofactor Pasha/DGCR8 to generate hairpin-structured premature-miRNAs (pre-miRNAs) with 60-70 nt (111). A single pri-miRNA transcript may generate more than one functional miRNA due to splicing (112). Then, Exportin-5 recognizes the 2-nucleotide overhang of the pre-miRNA and transports it from nucleus to the cytoplasm (113). In cytoplasm, the hairpin structure of pre-miRNAs is cleaved by DICER protein to form mature miRNAs, which are incorporated into an RNA-induced silencing complex (RISC) (114). Argonaute (AGO) proteins, the components of the RISC, guide mature miRNAs to specific target regions within mRNA transcripts, leading to mRNA degradation or translation blockage (106).

Dysregulation of miRNA often gives rise to multiple human diseases especially cancers. Abnormal expression of miRNAs is closely associated with cancer formation, progression, invasion, metastasis, and chemosensitivity (6). The complex roles of miRNAs as either tumor suppressors or oncogenes have been largely demonstrated. The correlation between miRNA-mediated autophagy and chemoresistance has been attracting a lot of interest. The fact that autophagy plays dual roles in chemoresistance provides a useful explanation on how miRNAs could reverse or facilitate chemoresistance through regulating autophagy. Since the protective mechanism of autophagy is the majority of cases to trigger tumor chemoresistance, inhibition of such autophagy by miRNAs may pave the way to combat chemoresistance. However, miRNA mediated-chemosensitivity by pro-death autophagy also possesses great value. Various tumor suppressive miRNAs are down-regulated in drug resistant cancer cells compared with sensitive ones, indicating that rejuvenation of these miRNAs may reverse chemoresistance by inhibiting protective autophagy or facilitating autophagic cell death. On the contrary, suppression of oncogenic miRNAs to modulate autophagy is another strategy for reversal of chemoresistance. In this section, we discuss the involvement of miRNAs in chemoresistance/chemosensitivity from different perspectives regulation of autophagy.

### Overexpression of MiRNA Reverses Chemoresistance by Inhibiting Autophagy

**Figure 1:**
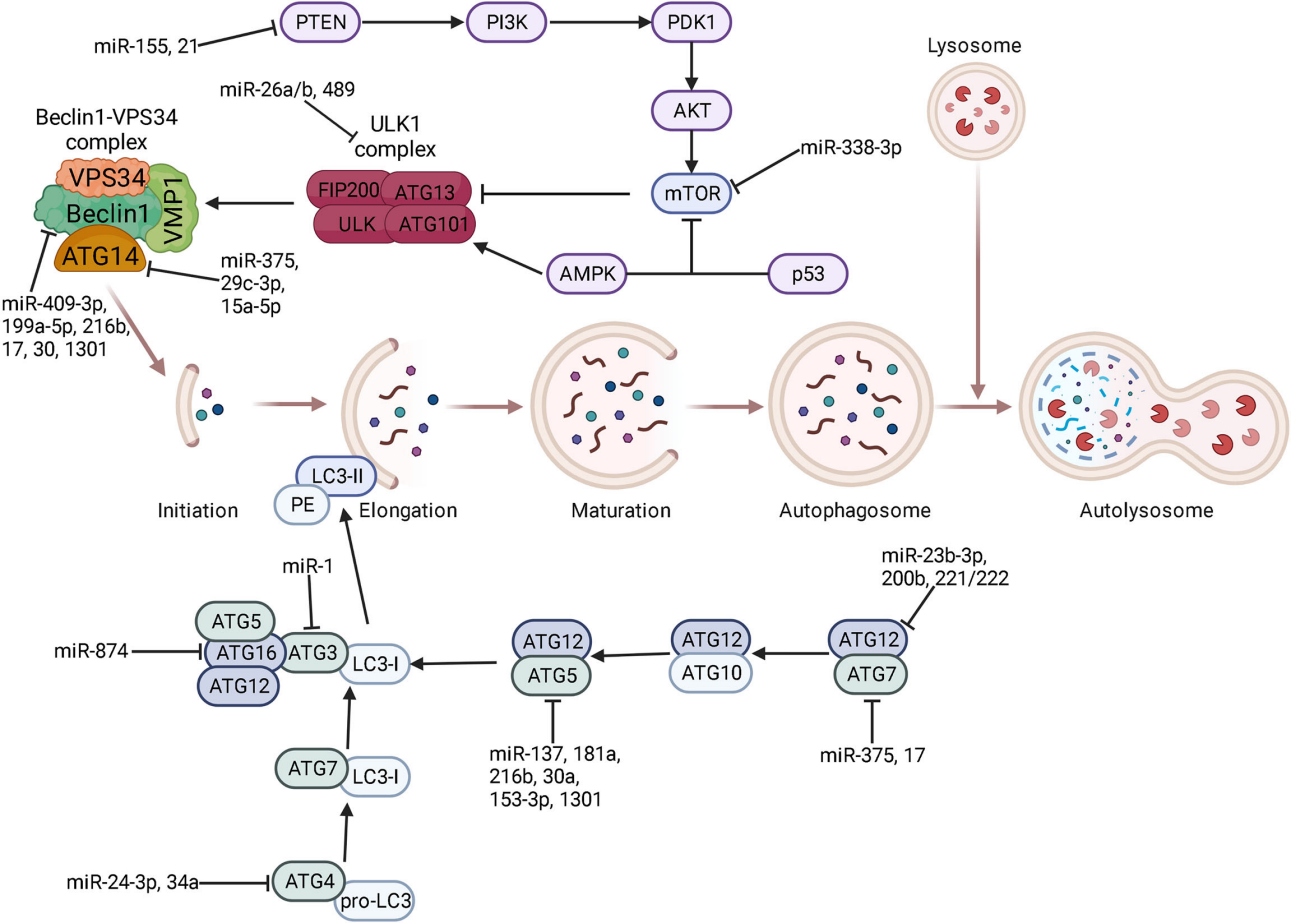
The regulatory role of miRNAs on each stage of autophagy. Core proteins and signaling pathways are related to each stage of autophagy including phagophore initiation and elongation, autophagosome maturation, and lysosomal fusion. Some key miRNAs target autophagy-related genes at the post-transcriptional level to participate in every stage of autophagy. ⊥ indicates an inhibitory effect and → indicates a promoting effect.

The process of autophagy is tightly regulated by ATGs, therefore targeting ATGs represent a promising strategy for reversal of drug resistance. Numerous ATGs are reported to be direct targets of miRNAs (**Figure 1**). For example, the 3’-UTR of ATG5 mRNA can be bound by miR-137 (115), miR-181a (116), miR-216b (117), miR-30a (118), and miR-153-3p (119) to facilitate ATG5 mRNA degradation, thus sensitizing various cancers, such as pancreatic cancer, gastric cancer, melanoma, chronic myelogenous leukemia, and non-small cell lung cancer (NSCLC), to chemotherapeutic agents by inhibiting protective autophagy. In addition to ATG5, Bcl-2 is a direct target of miR-153-3p in combating resistance to Imatinib in chronic myeloid leukemia (120). Similarly, miR-375 targets ATG7 (121) and ATG14 (122) to mediate chemosensitivity of HCC to sorafenib. The above studies indicated that a single miRNA can target different genes, likewise a specific gene is regulated by multiple miRNAs. Beclin-1, also known as ATG6, is a component of the PI3K complex which mediates vesicle-trafficking processes in autophagy (123). Targeting Beclin-1 by miR-409-3p (124), miR-17 (125), miR-216b (117), miR-17-5p (126), and miR-199a-5p (127) appears to reverse chemoresistance by inhibiting autophagy in different types of cancers. The interaction between miR-30 family and Beclin-1 has been revealed in recent years. It is reported that miR-30 or its homology miR-30a, miR-30a-5p binds to Beclin-1 mRNA to block autophagy-induced chemoresistance in chronic myeloid leukemia (118, 128), gastric cancer (129), osteosarcoma (130), small cell lung cancer (SCLC) (131) and other variety of cancers (132). A clinical study on Egyptian patients with chronic myeloid leukemia also confirmed this result (133). It was found that a single miRNA in different cancer types may share a common mechanism in mediating chemoresistance/chemosensitivity. Additionally, other ATGs are directly targeted by miRNAs, i.e. miR-541 targeting ATG 2A (134), miR-24-3p targeting ATG4A (135), miR-1 targeting ATG3 (136), miR-23b-3p and miR-200b targeting ATG12 (83, 137), miR-874 targeting ATG16L1 (138) and miR-34a targeting ATG4B (139). Therefore, overexpression of these miRNAs may result in sensitization of chemotherapeutic drugs in cancer treatment. Furthermore, miRNAs can also regulate ATGs indirectly. For instance, MiR-29c-3p targets FOXP1 to downregulate ATG14, leading to sensitization of ovarian cancer cells to cisplatin treatment by inhibiting autophagy (140).

High mobility group box 1 (HMGB1) is a highly conserved DNA-binding nuclear protein which regulates various DNA-related activities such as replication, transcription, and repair (141). Abundant studies have confirmed the involvement of HMGB1 in multiple hallmarks of cancers, making HMGB1 a promising target to combat tumor progression, invasion, metastasis, and chemoresistance (142). As a key regulator of autophagy, HMGB1 promotes drug resistance of a number of cancers including osteosarcoma (143), lung adenocarcinoma (144), and leukemia (145) by activating protective autophagy following pharmacotherapy. Some investigators have attempted to reveal the association between miRNAs and HMGB1. They found that HMGB1 was targeted by miR-22, miR-218, miR-26a, miR-34a, miR-129-5p, and miR-142-3p to sensitize osteosarcoma, endometrial carcinoma, melanoma, retinoblastoma, breast cancer, NSCLC to chemotherapeutic agents through inhibiting autophagy (146–151). HMGN5 is another member of the HMG box family involved in oncogenesis and tumor progression. Meng and his colleagues conducted a series of experiments to elucidate the HMGN5-associated chemoresistance. Their work revealed that HMGN5-mediated autophagy contributes to chemoresistance in osteosarcoma. Targeting HMGN5 by miR-140-5p sensitizes osteosarcoma cells to chemotherapy, suggesting a potential application of miR-140-5p in the prognosis and treatment of chemoresistant cancers (152).

Other core autophagic components, regulators, or signaling pathways also associated with the mechanisms of miRNA-mediated chemosensitivity. For example, RAB family, the largest subfamily of Ras, consists of more than 60 small GTPases. RABs play essential roles in membrane traffic including autophagosome formation (153). Xu et al. found that high miR-541 expression potentiates the response of HCC to sorafenib treatment by targeting RAB1B (134). Additionally, miR-148a-3p inhibits the cytoprotective autophagy by suppressing RAB12 to enhance cisplatin cytotoxicity in gastric cancer (154). As a key initiator of autophagy, ULK1 is an attractive target for cancer treatment. The 3ʹ-UTR of ULK1 was reported to contain binding sites for miR-26a/b and miR-106a. Overexpression of miR-26a/b enhances the sensitivity of HCC to doxorubicin (Dox) and promotes apoptosis both *in vitro* and *in vivo* by inhibiting autophagy (155). Similarly, ectopic expression of miR-106a resulted in significant tyrosine kinase inhibitor (TKI)-induced cell death in lung adenocarcinoma compared to control transduced cells (156). Moreover, miR-489 overexpression inhibits ULK1 to suppress autophagy, thus sensitizing breast cancer cells to DOX (157). FOXO3a is a multifaceted transcription factor which guides autophagy directly or indirectly (158). A research by Zhou et al. revealed that FOXO3a is a direct downstream target of miR-223. Overexpression of miR-223 or agomiR-223 contributes to the enhancement of doxorubicin sensitivity in HCC (159). Furthermore, Wingless-type MMTV integration site family member 2 (WNT2) belongs to the WNT family which is evolutionarily conserved (160). The foremost roles of the Wnt/β-catenin signaling pathway in tumorigenesis and tumor progression have been well established especially in the aspects of cancer invasion and migration, whereas little is known about WNT and autophagy (161). Chen et al. found that overexpression of miR-199a/b-5p inhibits its direct target WNT2 and downstream signaling to influence autophagy formation, resulting in enhanced efficacy of Imatinib treatment in chronic myeloid leukemia (162). Overall, induction of autophagy following chemotherapy confers the survival mechanism of cancer cells. Overexpression of some tumor suppressor miRNAs to block autophagy has become a useful strategy to enhance chemosensitivity *via* different molecular pathways. See **Table 1** for details.

**Table 1:** Overexpression of miRNA combat chemoresistance by regulating autophagy.

miRNA	Effect on autophagy	Cancer type	Resistant to	Targets	Ref
**miR-541**	Inhibition	HCC	Sorafenib	ATG2A, RAB1B	(134)
**miR-375**			Sorafenib	ATG7	(121)
**miR-223**			Doxorubicin	FOXO3a	(159)
**miR-26a/b**			Doxorubicin	ULK1	(155)
**miR-375**			Sorafenib	ATG14	(122)
**miR-125b**			Oxaliplatin	EVA1A	(163)
**miR-153-3p**	Inhibition	NSCLC	Gefitinib	ATG5	(119)
**miR-142-3p**			Adriamycin, Cisplatin	HMGB1	(149)
**miR-1**			Cisplatin	ATG3	(136)
**miR-129-5p**	Inhibition	Breast cancer	Taxol	HMGB1	(150)
**miR-451a**			Tamoxifen		(164)
**miR-214**			Tamoxifen, Fulvestrant	UCP2	(165)
**miR-27a**			Paclitaxel, Doxorubicin		(166)
**miR-489**			Doxorubicin	ULK1	(157)
**miR-24-3p**	Inhibition	SCLC	Etoposide, Cisplatin	ATG4A	(135)
**miR-30a-5p**				Beclin-1	(131)
**miR-495-3p**	Inhibition	Gastric cancer	Multidrug	GRP78	(167)
**miR-23b-3p**			Multidrug	ATG12 and HMGB2	(83)
**miR−30**			Multidrug	Beclin-1	(129)
**miR-874**			Multidrug	ATG16L1	(138)
**miR-181a**			Cisplatin	ATG5	(116)
**miR-148a-3p**			Cisplatin	AKAP1 and RAB12	(154)
**miR-29c**	Inhibition	Pancreatic cancer	Gemcitabine	USP22	(168)
**miR-137**			Doxorubicin	ATG5	(115)
**miR-101**	Inhibition	Osteosarcoma	Doxorubicin		(169)
**miR-22**			Cisplatin, Doxorubicin	HMGB1, MTDH	(146, 170, 171)
**miR-199a-5p**			Cisplatin	Beclin-1	(127)
**miR-30a**			Doxorubicin	Beclin-1	(130)
**miR-140-5p**			Multidrug	HMGN5	(152)
**miR-17**	Inhibition	Lung cancer	Paclitaxel	Beclin-1	(125)
**miR-106a**			Saracatinib, Dasatinib	ULK1	(156)
**miR-17-5p**			Paclitaxel	Beclin-1	(126)
**miR-200b**			Docetaxel	ATG12	(137)
**miR-26a**	Inhibition	Melanoma	Dabrafenib	HMGB1	(151)
**miR-216b**			Vemurafenib	Beclin-1, UVRAG, ATG5	(117)
**miR-409-3p**	Inhibition	Colon cancer	Oxaliplatin	Beclin-1	(124)
**miR-22**	Inhibition	Colorectal cancer	5-FU	BTG1	(172)
**miR-218**			Multidrug	YEATS4	(173)
**miR-34a**			Oxaliplatin	Smad4	(174)
**miR-199a/b-5p**	Inhibition	Chronic myeloid leukemia	Imatinib	WNT2	(162)
**miR- 30A**				Beclin-1, ATG5	(118, 128)
**miR-153-3p**				Bcl-2	(120)
**miR-17**	Inhibition	Glioblastoma	Temozolomide	ATG7	(175)
**miR-93**					(176)
**miR-218**	Inhibition	Endometrial carcinoma	Paclitaxel	HMGB1	(148)
**miR-30a**	Inhibition	Renal cell carcinoma	Sorafenib	Beclin-1	(177)
**miR-30a**	Inhibition	Various types of cancer	cis-DDP, Taxol	Beclin-1	(132)
**miR-34a**	Inhibition	Prostate cancer	Topotecan, Doxorubicin	ATG4B	(139)
**miR-29c-3p**	Inhibition	Ovarian cancer	Cisplatin	FOXP1/ATG14	(140)
**miR-199a-5p**	Inhibition	Acute myeloid leukemia	Adriamycin	DRAM1	(178)
**miR-34A**	Inhibition	Retinoblastoma	Vincristine, Etoposide, Carboplatin	HMGB1	(147)
**miR-15a/16**	promotion	Cervical carcinoma	Camptothecin	Rictor	(179)
**miR-181**	promotion	NSCLC	Cisplatin	PTEN/PI3K/AKT	(180)
**miR-193b**	promotion	Oesophageal cancer	5-FU	Stathmin 1	(181)
**miR-519a**	promotion	Glioblastoma	Temozolomide	STAT3/Bcl2	(182)

### Overexpression of MiRNA Reverses Chemoresistance by Promoting Autophagy

The inactivation of apoptosis pathway following chemotherapy contributes to the development of drug resistance. Hence, alternative types of cell death to combat chemoresistance have attracted increasing attention. Opposite to cytoprotective autophagy, excessive autophagy promotes autophagic cell death during chemotherapy (183). It has been reported that some miRNAs trigger autophagic cell death in drug resistant cancers by repressing important upstream signals of autophagy pathway. These cases are few but of great significance. In an investigation into chemosensitivity of cervical carcinoma, Huang et al. found that miR-15a and miR-16 directly targets Rictor to attenuate the phosphorylation of mTORC1 and p70S6K. As a consequence, miR-15a/16 dramatically enhances chemotherapeutic efficacy of camptothecin towards cervical carcinoma partly due to autophagy-induced inhibition of cell proliferation (179). Similarly, another research revealed that in cisplatin-resistant NSCLC, downregulation of miR-181 correlates with reduced autophagy and apoptosis. MiR-181 overexpression restored LC3 and ATG5 protein by triggering PTEN/PI3K/AKT/mTOR signaling pathway, therefore promoting apoptosis in cisplatin-resistant NSCLC (180). These two studies emphasize mTOR as the key regulator in miRNA-induced autophagic cell death. In a study of miR-193b, it is reported that overexpression of miR-193b significantly enhances the cytotoxicity of 5-FU to oesophageal cancer cells, which is mediated by elevated autophagic flux rather than apoptosis. Although the exact targets of miR-193b are unknown, target prediction analysis suggests that stathmin 1 might be involved in this process (181). Additionally, another research demonstrated that miR-519a increased the sensitivity of glioblastoma to temozolomide through induction of autophagy by targeting STAT3/Bcl-2/Beclin-1 signaling pathway. These results provide an effective therapeutic strategy of drug combination for glioblastoma treatment (182). See **Table 1** for details.

### Inhibition of MiRNA Reverses Chemoresistance by Enhancing Autophagy

The expression of some autophagy inhibitory miRNAs was significantly increased in drug resistant cancer cells compared with their parental cells, indicating the possible mechanism of miRNAs-mediated chemoresistance by autophagy. Hence, silencing these oncogenic miRNAs may increase the sensitivity of drug resistant cancer cells to chemotherapeutic agents by inducing autophagic cell death. For example, miR-1301 promoted the proliferation of cisplatin-resistant ovarian cancer cells by inhibiting ATG5 and Beclin1, indicating that targeting miR-1301 is an effective approach to reverse cisplatin resistance by inducing autophagy (184). Another research demonstrated that miR-487b-5p at high level may be a potential biomarker of acquired Temozolomide resistance in lung cancer cells. MiR-487b-5p directly targets LAMP2 to block autophagy thus mediating Temozolomide resistance. In consequence, miR-487b-5p has been regarded as a chemotherapeutic target in the treatment of TMZ-resistant lung carcinoma by enhancing autophagy (185). Furthermore, inhibitions of miR-221/222 induced extended autophagy and cell death of multiple myeloma cells by enhanced autophagy *via* ATG12 and p27 upregulation (186). A recent study found that miR-15a-5p was overexpressed in chemoresistant acute myeloid leukemia (AML) patients compared with chemosensitive patients treated with daunorubicin and cytarabine. The upregulation of miR-15a-5p decreased daunorubicin-induced autophagy by targeting ATG9a, ATG14, GABARAPL1, and SMPD1, thus resulting in attenuating cell sensitivity to daunorubicin. This finding indicated that inhibition of miR-15a-5p may sensitize AML to daunorubicin by enhancing autophagy (187). MiR-21 contributes to the tamoxifen (TAM) and fulvestrant (FUL) resistance of breast cancer by inhibiting autophagy. Yu et al. found that silencing miR-21 increased the sensitivity of ER^+^ breast cancer cells to TAM or FUL by triggering autophagic cell death. Phosphatase and tensin homolog (PTEN) is the potential target of miR-21 to regulate autophagy by affecting downstream PI3K-AKT-mTOR signaling pathway (188). As mentioned above, PI3K-AKT-mTOR is the core negative regulator of autophagy (189). The activation of PI3K-AKT-mTOR by miR-21 reduces the efficacy of cisplatin on gastric cancer cells through autophagy inhibition (190). Similarly, a study by He et al. verified the role of miR-21 in mediating sorafenib resistance of HCC cells by inhibiting autophagy. Anti-miR-21 oligonucleotides re-sensitized sorafenib-resistant HCC cells by promoting autophagy *via* the PTEN/AKT signaling pathway (191). These studies show that the connections of miR-21 and PTEN-PI3K-AKT-mTOR have been well established in drug resistance of some cancer types. Meanwhile, the autophagy inhibition by miR-155 through PTEN-PI3K-AKT-mTOR signaling pathway to mediate adriamycin resistance of osteosarcoma has been proposed (192). To conclude, targeting miR-21 or miR-155 may restore PTEN to inhibit PI3K-AKT-mTOR signaling pathway, thus triggering autophagic cell death to overcome chemoresistance. Seca et al. found that autophagy enhancement by miR-21 inhibition decreases the expression of pro-survival genes such as Bcl-2, thus sensitizing leukemia cells to chemotherapeutic drugs (193). Furthermore, recent studies confirmed that the downstream biological effects following autophagy inhibition may benefit cancer survival. The suppression of autophagy by miR-3127-5p results in activation of STAT3 signaling pathway, which stimulates programmed death-ligand 1 (PD-L1) and subsequently mediates immune evasion of cancer cells (194). As a consequence, targeting miR-3127-5p to facilitate autophagy may aid in immune escape dismission and chemoresistance reversal. See **Table 2** for details.

**Table 2:** Inhibition of miRNA combat chemoresistance by regulating autophagy.

miRNA	Effect on autophagy	Cancer type	Resistant to	Targets	Ref
**miR-1301**	Inhibition	Ovarian cancer	Cisplatin	ATG5 and Beclin1	(184)
**miR-487b-5p**		Lung cancer	Temozolomide	LAMP2	(185)
**miR-155**		Osteosarcoma	Adriamycin	PTEN	(192)
**miR-221/222**		Multiple myeloma	Dexamethasone	ATG12	(186)
**miR-3127-5p**		NSCLC	Cisplatin	STAT3	(194)
**miR-15a-5p**		Acute myeloid leukemia	Daunorubicin	ATG9a, ATG14, GABARAPL1, SMPD1	(187)
**miR-21**	Inhibition	Breast cancer	Tamoxifen, Fulvestrant	PTEN	(188)
		HCC	Sorafenib	PTEN/AKT	(191)
		Gastric cancer	Cisplatin	PI3K/AKT/mTOR	(190)
		Leukemia	Etoposide, Doxorubicin	Bcl-2	(193)
	Promotion	Colorectal cancer	Topoisomerase	proteasome pathway	(195)
**miR-138**	Promotion	Glioblastoma	Temozolomide	BIM	(196)
**miR-140-5p**		Osteosarcoma	Doxorubicincisplatin	IP3K2	(197)
**miR-155**			Doxorubicincisplatin		(198)
**miR-338-3p**		p53 mutant colon cancer	5-Fu	mTOR	(199)
**miR-7-5p**		Cervical cancer	Cisplatin	PARP-1, Bcl-2	(200)
**miR-223**		NSCLC	Cisplatin	FBXW7	(201)

### Inhibition of MiRNA Reverses Chemoresistance by Inhibiting Autophagy

The levels of some miRNAs are positively correlated with cytoprotective autophagy and drug resistance following chemotherapy, therefore targeting these upregulated miRNAs in drug resistant cancer cells may restore chemosensitivity by inhibiting autophagy. MiR-138 was confirmed to be associated with glioblastoma cell survival and resistance to TMZ by inducing pro-survival autophagy which negatively correlates with BIM, the direct target of miR-138. Hence, targeting miR-138 may represent a novel strategy to overcome temozolomide resistance in glioblastoma by inhibiting autophagy (196). Interestingly, the role of miR-21 in autophagy regulation is controversial. Contrary to what is mentioned in previous section, miR-21-5p enhances pro-survival autophagic flux following inhibition of proteasome pathway to mediate drug resistance to topoisomerase inhibitors in colorectal cancer (CRC). Therefore, miR-21-5p could be a potential target for reversing drug resistance in CRC (195). Moreover, miR-338-3p confers resistance to 5-FU in p53 mutant colon cancer through mTOR downregulation-induced autophagy, indicating that targeting miR-338-3p is a promising strategy to overcome 5-FU resistance in p53 mutant colon cancer (199). Additionally, miR-140-5p and miR-155 promote the chemotherapy-induced autophagy to mediate drug resistance in osteosarcoma. Inositol 1,4,5-trisphosphate kinase 2 (IP3k2) was reported to be a direct target of miR-140-5p (197, 198). Similarly, miR-7-5p promotes autophagy *via* suppression of Bcl-2 to mediate cisplatin resistance in cervical cancer (200). MiR-223 directly targets FBXW7 thus promoting autophagy and rendering NSCLC cells resistant to cisplatin (201). Thus, targeting the above mentioned miRNAs could provide potential approach to combat chemoresistance. See **Table 2** for details.

## Targeting miRNA/Autophagy Axis to Combat Chemoresistance

Since miRNAs play vital roles in chemoresistance and chemosensitivity *via* regulating autophagy, miRNA-based strategies including either miRNA inhibition or miRNA restoration have been proposed in cancer therapy. The rapid development of miRNA-based interventions, such as miRNA mimics, anti-miRNA oligonucleotides, miRNA sponges, and small molecule inhibitors has been witnessed in the last decade. Some of these agents are in different phases of clinical trials (202). The first miRNA-based therapy for cancer is MRX34, a miR-34 mimic designed to target Wnt signaling and tumor metastasis (203). In this section, we demonstrate some genetic or pharmacological interventions targeting miRNA to combat chemoresistance by modulating autophagy.

Isoliquiritigenin (ISL), a natural flavonoid isolated from the root of licorice, has been used for the treatment of inflammation, platelet aggregation, cancer, and cardiac injury for centuries (204). A research by Wang et al. revealed that ISL targets miR-25 to trigger autophagic cell death by increasing ULK1 expression in MCF-7/ADR cells, which provides evidence for ISL as a natural autophagy inducer to increase breast cancer chemosensitivity (205). Apigenin is a flavonoid with anti-proliferative properties against a broad spectrum of cancers (206). Apigenin can significantly upregulate miR-520b which targets ATG7 to block protective autophagy, thus sensitizing HCC cells to doxorubicin (207). Propofol is an intravenous sedative-hypnotic agent used in surgery. A growing number of studies have revealed the anti-tumor effect of propofol against different cancer types (208, 209). LncRNA MALAT1 targets miR-30e to facilitate autophagy *via* ATG5 upregulation. The downregulation of lncRNA MALAT1 by propofol results in inhibiting autophagy and promoting gastric cancer cells sensitive to cisplatin (210). A recent study demonstrated that rutin, the main component of *Potentilla discolor* Bunge, reverses sorafenib resistance by inhibiting autophagy through the BANCR/miRNA-590-5P/OLR1 axis in HCC (211). Furthermore, it is urgent to look for efficient miRNA delivery system for miRNA mimics that can’t enter cells efficiently on its own. Based on the novel drug delivery system, miR-375 and sorafenib were co-loaded into calcium carbonate nanoparticles with lipid coating (miR-375/Sf-LCC NPs). As an inhibitor of autophagy, miR-375 enhances cytotoxicity of sorafenib both *in vitro* and *in vivo* by targeting ATG7, thus producing potent anti-tumor effect to combat sorafenib resistance (121).

## Conclusion and Perspectives

**Figure 2:**
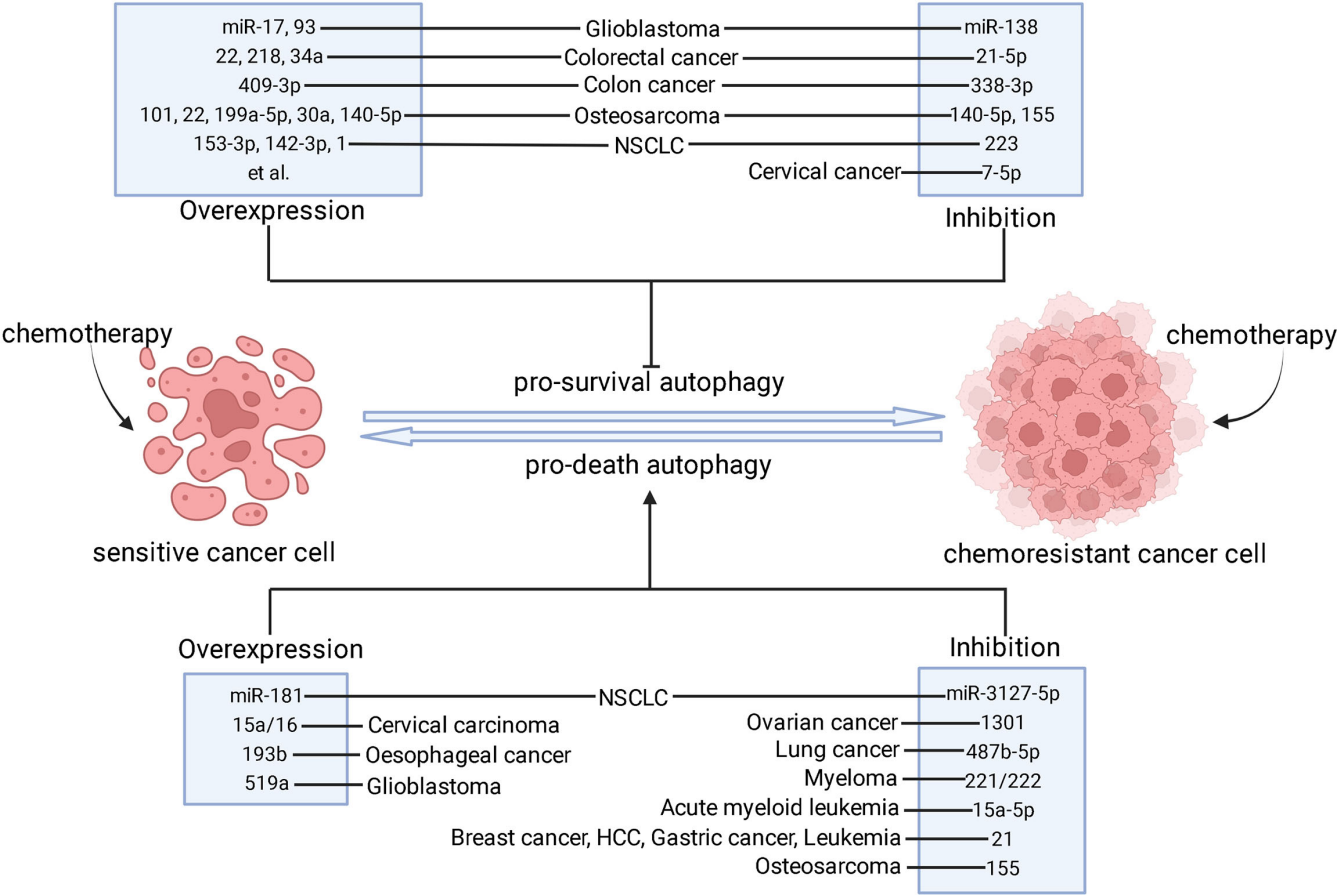
The strategies of modulating miRNAs to combat chemoresistance through autophagy. After chemotherapy is applied, sensitive cancer cells mainly undergo apoptotic cell death process whereas chemoresistant cancer cells fail to respond to chemotherapeutics. The pro-survival autophagy contributes to the development of chemoresistance. However, pro-death autophagy can be used as an alternative cell death mechanism in apoptosis-inactive cancer cells to re-sensitize them. Based on these facts, inhibition of pro-survival autophagy and induction of pro-death autophagy may result in chemoresistance reversal, which can be done by overexpression or inhibition of these miRNAs in different types of cancer. ⊥ indicates an inhibitory effect and → indicates a promoting effect.

This review demonstrated that miRNAs, as epigenetic factors of autophagy, play a pivotal role in cancer chemoresistance. Various types of cancers develop resistance to chemotherapeutic drugs through complex regulatory mechanisms of miRNAs by targeting different genes at every stage of autophagy. Due to the paradoxical effects of autophagy in chemoresistance, there is an urgent need to understand the interactions between miRNA-mediated autophagy and chemoresistance, which may provide evidence for development of novel miRNA-based therapy. As mentioned above, altered expression of miRNAs can trigger chemoresistance or chemosensitivity through pro-death or pro-survival autophagy during chemotherapy. Hence, inhibiting miRNA function or restoring miRNA expression is a possible approach for combating chemoresistance (**Figure 2**). Genetic interventions targeting miRNAs such as miRNA mimics, miRNA sponges, anti-miRNA oligonucleotides are useful approaches (212). The pharmacological interventions such as small molecule compound or active ingredient can also be used to target miRNA to overcome chemoresistance. In addition, the upstream molecular pathway regulating miRNA/autophagy axis can also be the potential targets for chemoresistance reversal. LncRNAs, circRNAs, and proteins are the major upstream mediators of miRNA/autophagy axis (213, 214). The complex regulatory network of upstream factors on miRNA/autophagy axis necessitates further research.

MiRNA-based therapy as an adjuvant to immunotherapy and targeted therapy is highly feasible. MiRNA-based therapies may aid in the four principal cancer immunotherapy approaches including immune checkpoint blockade, cancer vaccines, cytokine therapy, and adoptive cell therapy (215). According to the study of Howell et al, the miR-31 inhibits CD8^+^ T cell function, leading to a substantial block to anti-tumor immunity. Hence, they proposed that miR-31 inhibitor combined with PD-1 inhibitor may prevent T cell from exhaustion and promote autoimmunity, thus displaying huge potential for cancer suppression (216). MiR-200 has also emerged as a potential therapeutic adjuvant for checkpoint inhibitors by acting on both immune and metastatic pathways *via* modulation of PD-L1 and EMT (217). Additionally, aberrant expression of miRNAs promotes resistance of different types of cancer to targeted therapy through multiple mechanisms. Therefore, the combination of miRNA-based therapy and targeted therapy may overcome the resistance of cancer cells to targeted drugs such as tyrosine kinase inhibitors and monoclonal antibody (218).

Recently, the delivery approaches for miRNAs including viral vector-, lipid-, inorganic material-, polymer-, cell-, and 3D scaffold-based approaches have emerged (219). The lipid-based delivery systems such as liposomes, lipid nanoparticles, and solid lipid nanoparticles (SLNs) have been widely used for introduction of miRNAs. With the development of nanoparticle delivery system, the introduction of miRNA turns out to be highly efficient in cancer therapeutics because these nano-miRNAs have a site specific action, which can deliver the miRNA or anti-miRNA directly to the transformed cells, thus reducing the unexpected toxicity in non-target cells (212). The viral vector delivery system also has high efficiency. However, the associated immunogenic responses and cytotoxicity limit the further application of these approaches respectively (220, 221). Currently, the safety concerns of miRNA therapy including off-target side-effects, toxicity, and carcinogenicity have become big challenges. Nowadays, less than 20 miR targeting molecules have entered clinical trials, and none progressed to phase III (219). Hence, further research is needed to promote the application value of miRNA therapy.

In conclusion, this review elucidated the microRNA-based strategies to combat cancer chemoresistance *via* regulating autophagy. We expect that patients will benefit from the improvement of chemotherapy efficacy through modulation of miR/autophagy axis in the future.

## Author Contributions

YZ and LD contributed to conception and design of the review. YL, LC, and JL drafted the manuscript. All authors contributed to the article and approved the submitted.

## Funding

This work was supported by the National Natural Science Foundation of China (No. 81803790), National Natural Science Foundation of Guangdong (No. 2020A1515011090) and the Project of Administration of Traditional Chinese Medicine of Guangdong Province of China (Grant no. 20200511205949).

## Conflict of Interest

The authors declare that the research was conducted in the absence of any commercial or financial relationships that could be construed as a potential conflict of interest.

## Publisher’s Note

All claims expressed in this article are solely those of the authors and do not necessarily represent those of their affiliated organizations, or those of the publisher, the editors and the reviewers. Any product that may be evaluated in this article, or claim that may be made by its manufacturer, is not guaranteed or endorsed by the publisher.
